# Connectivity within and among a Network of Temperate Marine Reserves

**DOI:** 10.1371/journal.pone.0020168

**Published:** 2011-05-23

**Authors:** Melinda A. Coleman, Justine Chambers, Nathan A. Knott, Hamish A. Malcolm, David Harasti, Alan Jordan, Brendan P. Kelaher

**Affiliations:** 1 New South Wales Marine Parks Authority, Batemans Marine Park, Narooma, Australia; 2 New South Wales Marine Parks Authority, New South Wales, Sydney, Australia; 3 Australian Genome Research Facility Ltd., Plant Genomics Centre, Glen Osmond, Australia; Institute of Marine Research, Norway

## Abstract

Networks of marine reserves are increasingly being promoted as a means of conserving marine biodiversity. One consideration in designing systems of marine reserves is the maintenance of connectivity to ensure the long-term persistence and resilience of populations. Knowledge of connectivity, however, is frequently lacking during marine reserve design and establishment. We characterise patterns of genetic connectivity of 3 key species of habitat-forming macroalgae across an established network of temperate marine reserves on the east coast of Australia and the implications for adaptive management and marine reserve design. Connectivity varied greatly among species. Connectivity was high for the subtidal macroalgae *Ecklonia radiata* and *Phyllospora comosa* and neither species showed any clear patterns of genetic structuring with geographic distance within or among marine parks. In contrast, connectivity was low for the intertidal, *Hormosira banksii*, and there was a strong pattern of isolation by distance. Coastal topography and latitude influenced small scale patterns of genetic structure. These results suggest that some species are well served by the current system of marine reserves in place along this temperate coast but it may be warranted to revisit protection of intertidal habitats to ensure the long-term persistence of important habitat-forming macroalgae. Adaptively managing marine reserve design to maintain connectivity may ensure the long-term persistence and resilience of marine habitats and the biodiversity they support.

## Introduction

Marine reserves are increasingly being promoted as a means of managing coastal resources and protecting marine biodiversity [1]–[4]. Hundreds of published studies worldwide have shown their success in achieving these goals [5]–[7]. Although individual marine reserves are often designed to meet specific conservation needs for species or habitats, networks of connected reserves are widely acknowledged to be an important tool for ensuring the long-term health and sustainability of marine environments [8], [9].

A key reason for designing networks of marine reserves is connectivity [10], [11]; the exchange of genetic material, species or resources within and among populations. This aspect of marine reserve planning is important for maintaining and restoring natural ecological processes as well as genetic diversity. Connectivity may also ensure the long-term persistence and resilience of populations under both current and future scenarios of anthropogenic change. The size, spacing and arrangement of protected areas relative to scales of dispersal and life history of organisms [12] combined with local and regional scale oceanography [13] and other environmental factors determines the extent to which areas chosen for protection are connected and contribute to conservation goals. Understanding scales of connectivity is also a key consideration in marine reserve planning because it can enhance predictions about population dynamics and the ability for population recovery or rehabilitation, as well as assists in identification of areas as important sources or sinks of propagule dispersal [14], [15].

Temperate marine reserves worldwide are often dominated by macroalgae that constitutes a major biogenic habitat. Macroalgal habitats play a key role in maintaining marine biodiversity because they support a complex array of associated organisms [16]–[18]. Designing marine reserves to ensure connectivity of such ecologically-important habitats is pertinent given anthropogenic stressors are driving significant declines in macroalgal habitats worldwide [19]–[23]. Further, increasing coastal development is limiting and fragmenting the availability of suitable habitat for macroalgae [24] which has cascading effects on associated biodiversity [25]. Ensuring that marine reserves adequately protect macroalgal habitats, including maintaining connectivity, is critical to the conservation of temperate marine biodiversity as a whole.

A network of marine reserves has been established along ∼800 km of the coast of New South Wales, Australia. Macroalgae constitute the dominant and most conspicuous biogenic habitats on rocky reefs within most of these reserves [26], [27] and support a substantial component of nearshore biodiversity. We characterise patterns of connectivity of 3 key species of habitat-forming brown macroalgae, *Ecklonia radiata* (C.Agardh) J.Agardh, *Phyllospora comosa* (Labillardière) C. Agardh and *Hormosira banksii* (Turner) Decaisne, within and among this network of temperate reserves. Given the strength of ocean currents along this coastline (the East Australian Current), we predict that connectivity will be reasonably high for all species, but variations will be correlated with differences in morphology and life history. Specifically, we predict that macroalgae possessing the ability for long-distance dispersal (e.g. gas-filled vesicles that facilitate rafting) will have greater connectivity than those that lack such structures. We discuss the implications of connectivity of these ecologically-important macroalgal habitats for marine reserve design on both local (within reserve) and regional scales (networks of reserves).

## Methods

**Figure 1:**
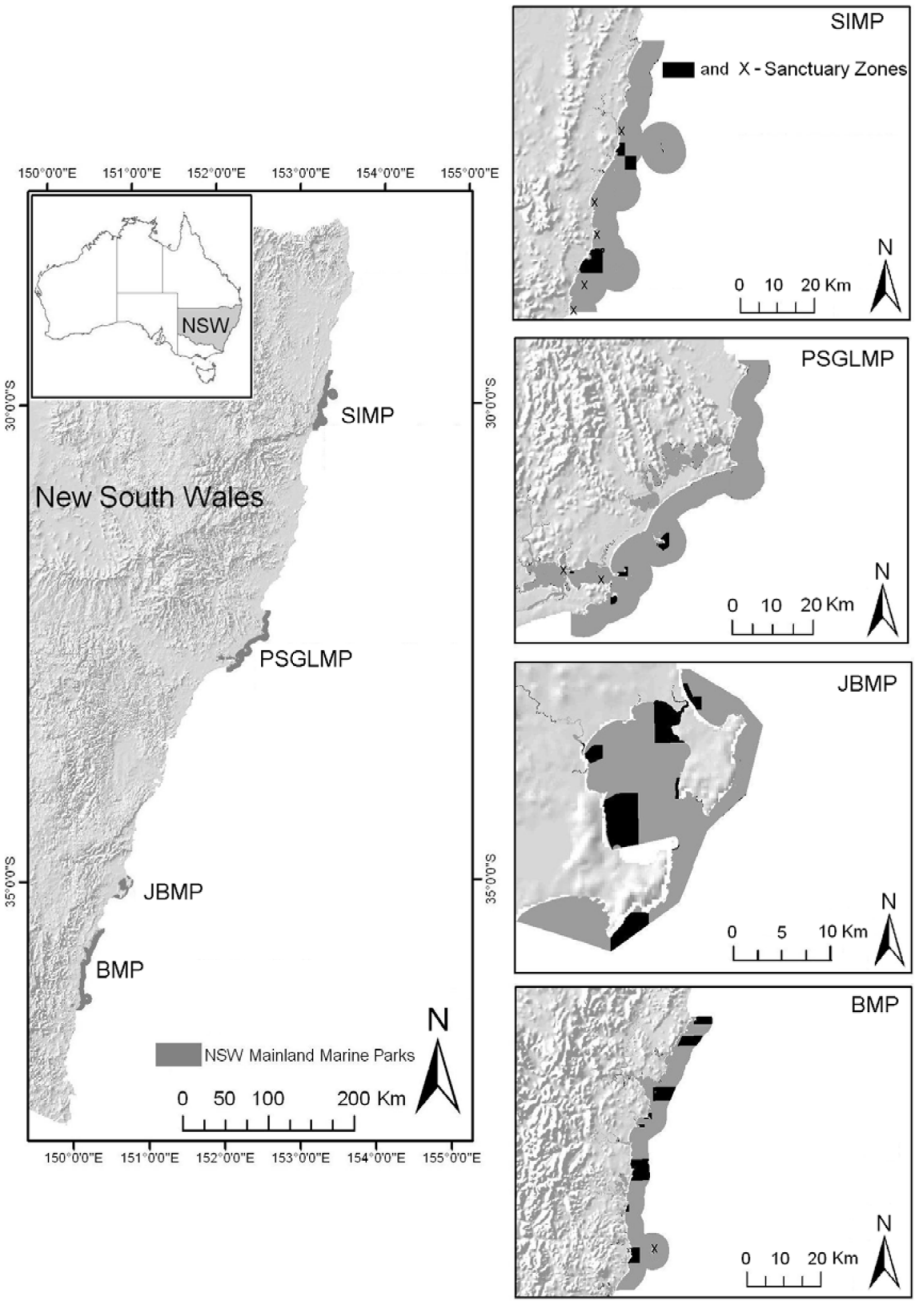
Map of New South Wales Marine Parks. Map showing marine parks and position of sanctuary zones that were sampled within each marine park. Small sanctuary zones are marked with an X.

Marine reserves in New South Wales provide protection on a hierarchy of spatial scales with replicate marine “parks” along the coast each containing a number of fully protected “reserves” or sanctuary zones (Fig. 1). We chose the 4 largest marine parks to characterise patterns of connectivity. From low to high latitudes these parks are Solitary Island Marine Park (SIMP; 20 years old and 72,000 Ha), Port Stephens-Great Lakes Marine Park (PSGLMP; 4 years old and 98,000 Ha), Jervis Bay Marine Park (JBMP; 8 years old and 22,000 Ha) and Batemans Marine Park (BMP; 4 years old and 85,000 Ha) (Fig. 1). Parks are between 150 (BMP and JBMP) and 740 km (BMP and SIMP) apart. Within each park, macroalgae were sampled from 5 to 9 geographically separated sanctuary zones (Fig. 1). Sanctuary zones (∼12 to 20% of each park) are no-take areas where development and anthropogenic impacts are limited via legislation. Populations of marine species within sanctuary zones were sampled because these zones are predicted to become increasingly more important to the long-term persistence of macroalgal forests due to diminished anthropogenic disturbance and decreased top-down grazing pressure [28]. Although there is no commercial harvesting of these macroalgal species anywhere in NSW, the public may collect small amounts of seaweeds within bag limits for non-commercial purposes outside of sanctuary zones, but the extent to which this activity occurs is minimal and we have never observed collection by the public. Sanctuary zones within each park ranged in size from 0.05 to 30 km^2^ and were different distances apart (1 to 21 km). Relevant permits for collection of algae at the 4 marine parks were obtained prior to collection.

We chose the 3 most abundant, perennial species of habitat-forming macroalgae to characterise patterns of connectivity; *Ecklonia radiata* (Laminariales, hereafter referred to as *Ecklonia*), the endemic *Phyllospora comosa* (Fucales, hereafter referred to as *Phyllospora*) and *Hormosira banksii* (Fucales, hereafter referred to as *Hormosira*). Each species represents the dominant form of biogenic habitat on intertidal or subtidal rocky reefs in temperate Australia and supports extremely diverse faunal assemblages [16], [18], [29]. The shallow subtidal *Phyllospora* and the intertidal *Hormosira* are both dioecious (obligate-outcrossers) with motile sperm and either sessile (*Phyllospora*) or negatively buoyant (*Hormosira*) eggs. In contrast, the kelp, *Ecklonia* has an alternations of generations life history. As with other laminarian macroalgae, sperm are likely capable of dispersal on small spatial scales (e.g. cm) [30] and zoospores have the potential to disperse further e.g. km [31], [32]. All 3 species are able to disperse via fertile drift material that is torn from the substratum during storms. In particular, *Phyllospora* and *Hormosira* possess gas-filled vesicles that may facilitate rafting long-distances [33], [34] relative to *Ecklonia* which does not possess such structures. All 3 species are considered cold temperate macroalgae and are nearing the northern limits of their distribution in northern NSW.

Portions of unfouled thalli of at least 32 mature individuals of each species were randomly collected from each sanctuary zone in 2009 and taken to the laboratory on ice. *Phyllospora* was only sampled from the southerly parks where it was most abundant (BMP and JBMP). Material was air dried, DNA was extracted and individuals were genotyped using 5 microsatellite loci for *Hormosira* and 7 for *Ecklonia* and *Phyllospora* [35], [36] and Coleman et al. (unpbl data). Prior to conducting statistical analyses, we checked data for null alleles using MICRO-CHECKER [37]. Patterns of genetic diversity within each sanctuary zone were compared using a number of different descriptive measures. The total number of alleles, observed (*H*_o_) and expected heterozygosities (*H*_e_) and *F*_IS_ (an estimate of inbreeding within populations) were estimated using GENETIX ver. 4.04 [38]. In addition, we tested for linkage disequilibrium and Hardy-Weinberg equilibrium at each locus and across all loci using FSTAT 1.2 [39].

We infer connectivity from estimates of genetic differentiation using Weir and Cockerham's *F*_ST_ estimates [40] generated in FSTAT 1.2, [39]. Although estimates of population differentiation (*F*_ST_) can reflect processes in addition to dispersal and connectivity (e.g. population history, population size, departures from an equilibrium model, etc) meta-analyses have shown that such additional processes rarely overwhelm estimates of dispersal and that *F*_ST_ is still an informative statistic for characterising connectivity [41]. Pairwise *F*_ST_ estimates were also estimated between parks and among sanctuary zones within parks. A sequential Bonferroni correction [42] was used when examining significance levels for pairwise tests. We did not assume random mating in these analyses, so genotypes (rather than alleles) were permuted. To determine the percentage of variation explained among and within marine parks, we conducted analyses of molecular variance (AMOVA) in ARLEQUIN ver. 3.00 [43]. We did not assume a stepwise mutation model. *P*<0.05 was used. Tests of isolation by distance were done via a Mantel test on pairwise *F*_ST_ and geographic distance matrices. This was done for all data and for each park separately using the program IBD [44]. For species exhibiting significant genetic differentiation, we identified potential first generation migrants using GeneClass 2 [45] as an indirect measure of past dispersal. Gene Class 2 uses Monte Carlo resampling techniques to compute the probability of an individual belonging to each given source population. Tests were done using Rannala and Mountain's (1997) Bayesian method of computing genotypes [46].

## Results

### Descriptive measures

There was no evidence of null alleles or linkage-disequilibrium for *Phyllospora* and all loci were in Hardy-Weinberg equilibrium. There were significantly more alleles over all loci in BMP relative to JBMP (ANOVA, 1, 13 d.f., *F* = 7.902, *P*<0.05). Both JBMP and BMP showed random mating with 13 out of 15 *F*_IS_ estimates being non-significant (Table 1).

**Table 1:** Descriptive genetic measures for each sanctuary zone and species.

		Total alleles			He			Ho			*F*_IS_		
Park	Sanctuary Zone	E	P	H	E	P	H	E	P	H	E	P	H
**SIMP**	**Arrawarra**	16	**-**	10	0.262	***-***	0.284	0.263	***-***	0.278	0.013	***-***	0.039
	**Baracoon**	15	**-**	13	0.251	***-***	0.347	0.244	***-***	0.257	0.045	***-***	*0.273*
	**Emerald**	16	**-**	12	0.215	***-***	0.117	0.232	***-***	0.081	−0.063	***-***	*0.319*
	**Flattop**	17	**-**	11	0.354	***-***	-	0.438	***-***	-	*−0.222*	***-***	**-**
	**Jones**	15	**-**	11	0.3221	***-***	0.287	0.438	***-***	0.290	*−0.344*	***-***	0.005
	**Split**	18	**-**	-	0.365	***-***	-	0.456	***-***	-	*−0.237*	***-***	**-**
	**Muttonbird Island**	-	**-**	11	-	***-***	0.267	-	***-***	0.269	-	***-***	0.006
**PSGLMP**	**Broughton**	19	-	12	0.273	*-*	0.350	0.269	-	0.420	0.030	*-*	−0.183
	**Cabbage**	15	**-**	11	0.220	*-*	0.324	0.190	*-*	0.250	0.152	*-*	*0.244*
	**Fingal**	17	**-**	12	0.228	***-***	0.325	0.169	***-***	0.348	*0.274*	***-***	−0.052
	**Halifax**	15	**-**	11	0.210	***-***	0.313	0.219	***-***	0.325	−0.023	***-***	−0.022
	**Piggys (B)**	-	**-**	10	-	***-***	0.265	-	***-***	0.175	-	***-***	*0.354*
	**Fame (B)**	-	-	10	-	-	0.329	-	-	0.240	-	-	*0.287*
**JBMP**	**Groper (B)**	18	31	15	0.332	0.502	0.377	0.373	0.558	0.368	−0.109	−0.096	0.045
	**Huskisson (B)(B)(B)**	17	20	17	0.337	0.296	0.485	0.425	0.326	0.389	*−0.246*	−0.087	*0.216*
	**Hyams (B)**	19	26	14	0.343	0.402	0.421	0.431	0.411	0.413	*−0.242*	−0.007	0.076
	**Hammer**	15	20	14	0.290	0.419	0.360	0.381	0.613	0.390	*−0.302*	*−0.394*	−0.065
	**Hare (B)**	16	18	14	0.300	0.314	0.333	0.363	0.299	0.368	−0.194	0.062	−0.087
	**Steamers**	17	29	15	0.331	0.485	0.383	0.369	0.512	0.401	−0.099	−0.039	−0.042
**BMP**	**Honeysuckle**	-	33	14	-	0.511	0.426	-	0.455	0.389	-	*0.124*	0.105
	**Montague Isld**	18	37	-	0.331	0.547	-	0.363	0.509	-	−0.080	0.085	-
	**Mullimburra**	17	32	14	0.329	0.518	0.378	0.338	0.491	0.416	−0.011	0.068	−0.082
	**Broulee**	17	23	15	0.303	0.515	0.489	0.370	0.513	0.490	*−0.205*	0.018	0.014
	**Guerilla**	-	32	14	-	0.529	0.399	-	0.536	0.425	-	0.003	−0.035
	**Tollgates**	19	28	14	0.316	0.486	0.299	0.307	0.478	0.316	0.043	0.032	−0.040
	**Brush Isld**	19	29	-	0.311	0.517	-	0.276	0.509	-	0.132	0.031	-
	**Fullers**	-	39	18	-	0.570	0.523	-	0.570	0.617	-	0.016	−0.162
	**Murramarang**	15	30	15	0.380	0.527	0.268	0.399	0.549	0.212	−0.025	−0.007	*0.225*

Total number alleles, expected (H_e_) and observed (H_o_) heterozygosity and *F*_IS_ (a measure if inbreeding within populations) for each sanctuary zone within Marine Parks. Numbers in italics are significant at *P*<0.01. E = *E. radiata*, H = *H. banskii* and P = *P. comosa*. Park abbreviations are as in materials and methods. Dashes indicate samples not collected. (B) indicates sanctuary zone located inside a bay.

For the kelp, *Ecklonia*, there was no evidence of null alleles but some evidence of linkage between Locus SSR11K32 and other loci. In addition, this locus was heterozygous in all sanctuary zones and was subsequently omitted from analyses. Some loci deviated from Hardy-Weinberg equilibrium at some sanctuary zones but there were no consistent patterns among loci or parks. Some sanctuary zones showed significantly negative *F*_IS_ estimates indicating excesses of heterozygotes while one (Fingal Bay in PSGLMP) was characterised by inbreeding/selfing (Table 1). There were no differences in the number of alleles among parks (ANOVA, *P*>0.05). Private alleles were only found in 2 sanctuary zones within BMP.

*Hormosira* showed no evidence of null alleles or linkage disequilibrium at any locus. Tests for Hardy-Weinberg equilibrium over all populations of *Hormosira* revealed that 3 loci deviated from random mating but patterns were variable among sanctuary zones (Table 1). Locus SSR2H12 was monomorphic in 50% of sanctuary zones, Locus SSR1H1 exhibited excesses of heterozygotes in 17% of sanctuary zones while SSR8H6 deviated from random mating in 67% of sanctuary zones but the direction of deviation was variable. At all other loci and sanctuary zones loci were in Hardy-Weinberg equilibrium. *F*_IS_ estimates for parks were mostly non-significant but 7 sanctuary zones were characterised by excesses of homozygotes indicating inbreeding (Table 1). The mean number of alleles per sanctuary zone was significantly greater in southerly parks (JBMP and BMP, 14 to 18 alleles) relative to northerly parks (SIMP and PSGLMP, 10 to 13 alleles; ANOVA, 3, 20 d.f. *F* = 17.10, *P*<0.001). Private alleles were found in 3 sanctuary zones within BMP and 1 within SIMP.

### Patterns of genetic structure within marine parks

**Figure 2:**
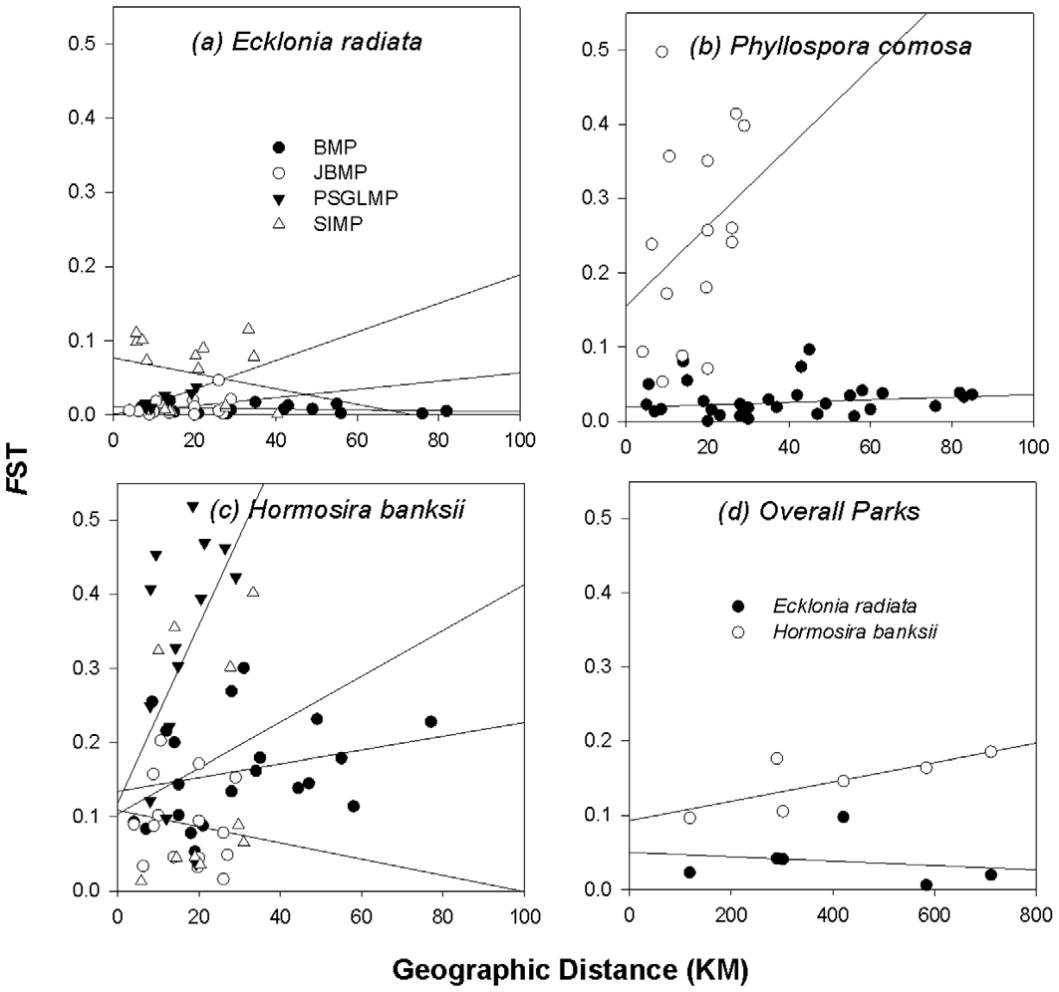
Relationship between geographic distance and genetic differentiation. Relationship between geographic distance and genetic differentiation. *F*_ST_ among sanctuary zones within each park and over all parks for each species.

Genetic differentiation of *Phyllospora* varied by orders of magnitude between parks with strong structure within JBMP and weak structure within the open coast BMP (Table 2). Within BMP less than one-eighth of pairwise tests between sanctuary zones were significant in contrast to JBMP where all pairs were statistically significant (Table 2). *Phyllospora* showed no correlation between genetic differentiation and geographic distance between sanctuary zones for either JBMP (Mantel test *Z* = 66.34, *r* = 0.321, *P*>0.05, Fig. 2) or BMP (Mantel test *Z* = 36.038, *r* = 0.169, *P*>0.05, Fig. 2). Further, in JBMP there was no obvious relationship between genetic diversity or differentiation of *Phyllospora* when sanctuary zones were located inside versus outside the Bay with mean *F*_ST_ estimates within the Bay (*F*_ST_ = 0.261) being similar to inside/outside comparisons (*F*_ST_ = 0.229).

**Table 2:** Summary of within park population structure for each species.

	*Ecklonia radiata*			*Hormosira banksii*			*Phyllospora comosa*		
	*F*_ST_	% SZ	IBD	*F*_ST_	% SZ	IBD	*F*_ST_	% SZ	IBD
**SIMP**	0.058	60	ns	*0.171*	90	ns	-	-	−
**PSGLMP**	0.016	17	+	*0.332*	73	+	-	-	−
**JBMP**	0.010	30	ns	*0.091*	27	ns	*0.238*	100	ns
**BMP**	0.012	0	ns	*0.151*	86	ns	*0.022*	12	ns
**ALL**	0.049	-	ns	*0.27*	-	+	*0.14*	-	−

*F*_ST_ estimates in italics are significant. % SZ refers to the percentage of pairwise tests between sanctuary zones within each park that were genetically different. IBD refers to patterns of isolation by distance (correlations between genetic differentiation and geographic distances) which could be either non-significant (ns) or positive correlations (+). Park abbreviations are as in materials and methods. IBD was not calculated over all marine parks for *Phyllospora* because only 2 marine parks were sampled.

For *Ecklonia*, there was insignificant population differentiation within most marine parks (Table 2). Despite this, pairwise tests did reveal that populations of *Ecklonia* in sanctuary zones within SIMP, PSGLMP and JBMP were sometimes significantly different (Table 2). There were no significant pairwise differences between sanctuary zones within BMP. Within SIMP, populations of *Ecklonia* within 2 sanctuary zones (Emerald and Flattop) differed from all others. The only park to exhibit a positive correlation between genetic differentiation and geographic distance between sanctuary zones was PSGLMP (Mantel test: *Z* = 1.264, *r* = 0.909, *P*<0.001, Table 2 and Fig. 2).

There were significant but varying levels of genetic differentiation within each park for *Hormosira* with the highest in PSGLMP and the lowest in JBMP (Table 2). Pairwise tests revealed that almost all sanctuary zones differed genetically from one another within each park, except within JBMP where only 27% of pairwise tests between sanctuary zones were significant (Table 2). Again, there was a significant relationship between genetic differentiation and geographic distance within PSGLMP (*Z* = 79.58, *r* = 0.52, *P*<0.05), but not within any other park (Fig. 2).

### Patterns of genetic structure across a network of marine parks

Estimates of genetic structure across the network of marine parks varied among species with high structure for *Hormosira* (*F*_ST_ = 0.27) and weaker structure for *Phyllospora* (*F*_ST_ = 0.14) and *Ecklonia* (*F*_ST_ = 0.049). For all species, all pairs of parks were genetically different indicating that dispersal is at least somewhat restricted on these scales, however, the magnitude of these differences varied between parks and species (Table 3).

**Table 3:** Pairwise *F*_ST_ estimates and percentage of significant pairwise tests between all marine parks for *Hormosira* and *Ecklonia*.

	SIMP	PSGLMP	JBMP	BMP
**SIMP**		*0.177* (100)	*0.164* (97)	*0.185* (100)
**PSGLMP**	*0.041* (58)		*0.105* (97)	*0.146* (100)
**JBMP**	*0.023* (58)	*0.098* (100)		*0.097* (76)
**BMP**	*0.006* (67)	*0.042* (92)	*0.020* (36)	

Pairwise *F*_ST_ estimates between all parks and percentage of significant pairwise tests between sanctuary zones from different parks (in parentheses) for *E. radiata* (bottom left of matrix) and *H. banksii* (top right). Significant values after the Bonferroni sequential correction are shown in italics. Park abbreviations are as in materials and methods.

Not surprisingly, pairwise tests between pairs of sanctuary zones from different parks were complex and varied among species. For *Phyllospora* and *Hormosira*, pairwise tests between pairs of sanctuary zones from different parks were almost always significant (93% for *Phyllospora*). Patterns were more complex for *Ecklonia* with varying levels of differentiation among different pairs of parks (Table 3).

AMOVA demonstrated that the amount of over all genetic variation explained at each spatial scale differed among species. For example, the proportion of over all genetic variation explained at the scale of the entire network of parks was 2 to 3% for *Ecklonia* and *Phyllospora* but 12% for *Hormosira* (Table 4). Similarly, the amount of over all genetic variation explained among sanctuary zones within parks varied from 2 to 17% (for *Ecklonia* and *Hormosira* respectively, Table 4). For all species, most genetic variability was explained among individuals within sanctuary zones (Table 4).

**Table 4:** Analysis of Molecular Variance (AMOVA) among all parks and among sanctuary zones within parks.

Source of variation	d.f.	SS	Variance component	Percentage of variation	
***Ecklonia radiata***					
Among Parks	3	29.95	0.024	3.07	*
Among sanctuary zones	18	31.60	0.016	2.08	**
Within sanctuary zones	1370	1008.99	0.735	94.86	***
Total	1391				
***Hormosira banksii***					
Among Parks	3	197.45	0.146	11.83	***
Among sanctuary zones	20	258.70	0.217	17.55	***
Within sanctuary zones	1510	1235.75	0.872	70.63	***
Total	1533				
***Phyllospora comosa***					
Among Parks	1	35.63	0.048	2.86	***
Among sanctuary zones	13	176.30	0.189	11.31	***
Within sanctuary zones	945	1358.59	1.438	85.83	**
Total	959				

*** = P<0.00001,

** = P<0.001,

* = P<0.05.

There was a significant relationship between genetic differentiation and geographic distance over all parks for *Hormosira* (Mantel test: *Z* = 383.99, *r* = 0.753, *P*<0.001) but not for *Ecklonia* (Fig. 2). For *Hormosira* we tested for first generation migrants to estimate putative levels of migration among sanctuary zones and parks. Despite low estimates of connectivity suggesting limited dispersal, in each park, many individuals were considered migrants (∼50 to 75%). Of these migrants, approximately half were likely sourced from a sanctuary zone within the same park (56–59%). Migrants with a likely source in another park were predominatly from adjacent parks.

## Discussion

Connectivity of habitat-forming macroalgae within and among a network of marine reserves along ∼800 km of Australia's temperate coastline varied greatly among key habitat-forming algal species. Connectivity across the network of parks was high for the large, subtidal macroalgae *Ecklonia* and *Phyllospora* [47] and low for the intertidal *Hormosira*. These patterns were generally reflected within each park and indicate that *Ecklonia* and *Phyllospora* are well served by the current system of marine reserves in place along the NSW coast.

Given the highly structured nature of populations of the intertidal *Hormosira*, the loss of localised populations may be particularly problematic for this species. Unlike the subtidal species, *Hormosira* has a more limited habitat range (midshore areas of the intertidal) and is more vulnerable to direct human interactions. For example, it has been demonstrated that cover can be substantially reduced from trampling, which has flow on impacts to associated communities [48], [29]. Moreover, this species also exists as a detached ecotype in estuarine mangrove forests (which was not sampled here) which may experience even higher genetic differentiation due to the more isolated nature of estuaries. Given the sensitivity of *Hormosira* to localised impacts and its highly structured populations, it may be warranted to revisit protection of intertidal habitats at Marine Park zoning plan reviews (every 5 to 10 years in New South Wales) to ensure long-term persistence of this important habitat-forming species. It should be noted, however, that the Sydney region has a few intertidal protected areas and aquatic reserves in which macroalgae are protected that augment protection afforded by Marine Parks.

### Ocean Currents and Connectivity

The East Australian Current (EAC) is the strongest of Australia's continental boundary currents reaching speeds of up to 3.6 m/s and generates a characteristic cyclonic and anticyclonic eddy field [49]. The EAC is likely to facilitate high connectivity of the subtidal macroalgae, *Ecklonia* and *Phyllospora*. These subtidal species broadcast spawn propagules directly into the ocean and dispersal may potentially occur over long distances. Further, fertile drift material that is torn from the substratum in storms has been found 100 s km away from its nearest source [34]. High connectivity appears to be a common pattern on the east coast of Australia and is found across a variety of marine organisms with planktonic propagules including anemones [50], cushion stars [51], urchins [52], abalone [53], fish [54], [55], *Phyllospora* and *Ecklonia* [47], [56].

The EAC is also characterised by seasonal variation in its strength and positioning allowing inshore, north-flowing counter currents that can facilitate bi-directional dispersal [56], [57]. In addition, cyclonic and anti-cyclonic eddies that are shed from the EAC periodically may promote non-linear dispersal of propagules as they are entrained in eddies and move on and offshore. This is likely to account for the genetic patchiness (lack of isolation by distance) seen in *Ecklonia* [56] and *Phyllospora* [47], as well as in many other species [49]–[54].

Given the high connectivity and lack of isolation by distance for *Ecklonia* and *Phyllospora*, the current system of marine reserves in place along this coastline is adequate to ensure that connectivity of these ecologically important species is maintained. Indeed, dispersal and mixing of genetic material along the NSW coastline appears to be substantial and conducive to resilience of these species to recolonise following perturbations. With predicted strengthening of the EAC with climate change over the next century [58] connectivity and dispersal may be further enhanced along this coastline. Nonetheless, concomitant warming ocean temperature and indirect effects from increased ocean acidification [59] may pose new problems (e.g. physiological stress, competition) for these temperate macroalgae.

### Connectivity, latitude and coastal topography

Latitude may play a role in structuring populations nearing their northern limit of distribution because of their often fragmented nature and variable population dynamics and ecology. The northern most marine park (SIMP) was the only place to exhibit significant genetic structure of *Ecklonia* with populations within 2 sanctuary zones differing from all others. This subtropical park is near the northern limit of distribution of *Ecklonia* and north of the main separation point of the EAC. In this park, kelp forests are interspersed with invertebrate and coral dominated habitat and genetic differentiation may thus arise due to the more fragmented nature of *Ecklonia* populations relative to their southerly counterparts. Further, relative to populations at higher latitudes, populations of kelp at lower latitudes often undergo intense grazing by herbivorous tropical fish that remove the entire canopy (pers. obs) and are known to exhibit an unusual annual life history [60] that may result in short-lived populations with high turnover and subsequent founder effects. Combined with the fact that recolonisation of kelp following pertubations at this northern limit of distribution may be compromised given the predominatly polewards flow of the EAC, populations at the limits of their distribution may warrant special consideration or conservation status [61].

Latitude also correlated with genetic diversity of *Hormosira* with greater allelic diversity (and expected heterozygosity) in parks at higher latitudes relative to parks at lower latitudes. Again, this pattern is perhaps not surprising given that northern NSW is the limit of distribution of this temperate alga and populations are likely to be smaller and more fragmented and experience greater population fluctuations as conditions near their physiological tolerances. Lower genetic diversity may confer a decreased ability to adapt to extreme conditions and populations at the limits of their distribution may be at greater risk of extinction under predicted scenarios of climate change. Multiple species of temperate macroalgae have already been observed to have shifted poleward on the coast of NSW [62] and this may be a consequence of a smaller gene pool and inability to adapt.

Coastal topography is known to influence patterns of connectivity of marine organisms and is likely to account for the contrasting patterns of connectivity of *Phyllospora* between the 2 marine parks in which it was sampled. Populations within the large embayment of JBMP displayed reasonably high population structure and are likely isolated from the main flow of the EAC by the protruding headlands and narrow entrance of this bay. Low connectivity as seen within JBMP appears to be atypical of the NSW coastline for this [55] and other species (see earlier references). It is likely that dispersal within the Bay, as well as between the Bay and open coast sites is restricted. Bays and estuaries can restrict gene flow, and populations within bays can sometimes act as sinks of genetic diversity [63]. Nevertheless, this pattern was not consistent among species indicating that other factors may be at play.

### Effects of morphology and habitat on connectivity

Contrary to predictions, the extent to which populations of macroalgae were connected was not related to morphology. The presence of gas-filled vesicles (e.g. *Phyllospora* and *Hormosira*) did not appear to greatly facilitate rafting and long-distance dispersal as evidenced by high genetic structure in *Hormosira* and, in some places, *Phyllospora* (JBMP). *Hormosira* has previously been shown to have the potential for long distance transport of fertile drift material that is torn from the substratum [64]. Despite many studies inferring this as a mechanism structuring large-scale genetic patterns in other algae [65], [47] it does not appear to be a significant driver of connectivity along the NSW coast for *Hormosira*. Nevertheless, approximately half of the *Hormosira* individuals sampled were classified as migrants that were predominatly from adjacent sanctuary zones in the same marine park suggesting that detached fertile individuals of this species must occasionally disperse on these spatial scales because fucoid fertilisation is generally rapid and zygotes presumably attach quickly to the substratum [66]. Low connectivity for *Hormosira* may be, however, related to the intertidal habitat of this species. Intertidal fucoid algae generally have limited dispersal potential [66] often reproducing during calm periods or low tide to minimise gamete dilution and ensure fertilisation success and subsequently attaching rapidly to the substratum. Mid to high intertidal invertebrates [67] and potentially algae, generally show greater genetic differentiation than counterparts in deeper habitats.

### Marine Reserve design and connectivity

In designing networks of marine reserves, the relationships between spatial distances among reserves and patterns of genetic differentiation are important considerations. Such patterns may help inform planning of the arrangement and spacing of reserves on both local and regional scales. There was no pattern of isolation by distance for the highly connected *Ecklonia* and *Phyllospora* but strong correlations for *Hormosira* over the entire network of parks. These patterns were consistent within parks except for *Hormosira* where positive correlations were restricted to 1 park (PSGLMP). The spatial arrangement and distances among reserves are therefore important considerations for ensuring connectivity of *Hormosira* along this coastline. Clearly, dispersal and subsequent connectivity in this species is often reliant on distances between populations or availability of rocky reef. Therefore, consideration must be given to the design of networks of marine reserves to ensure adequate protection of this species, particularly in light of increasing foreshore modification and development.

With networks of marine reserves being established in temperate regions worldwide there is a critical need for developing predictive models of dispersal and connectivity, particularly where little extant data exists. Generalising connectivity is problematic as spatial patterns of genetic structure can be vastly different among species and among coastlines and thus each may require specific conservation efforts. A classic example is *Ecklonia* which occurs throughout the temperate coastlines of Australia. Connectivity in this species is vastly different among Australia's eastern, southern and western coasts and differences correlate with the peak strength of continental boundary currents [56]. Thus, while the nature of the EAC may promote dispersal and facilitate the effectiveness of networks of marine protected areas on Australia's east coast, on the southern coastline for example, the weak Flinders Current promotes poor connectivity where spatial scale determines genetic structuring [68]. Networks of marine reserves must therefore be designed based on information of specific coastlines of interest relative to broadscale oceanography and species life-history.

Maintaining connectivity alone will not ensure the long-term persistence of macroalgal or other important marine habitats. With the synergistic effects of forecast climatic changes and increasing anthropogenic stressors there are likely to be large and dramatic effects on important macroalgal habitats [59] particularly at lower latitudes. While marine reserves may do little to halt warming oceans, they can lessen non-climatic stress thereby increasing the resilience of marine communities. This emphasises the importance of establishing protected areas where both top down (e.g. harvesting and fishing) and bottom up (pollution, development etc) stressors are limited. Such protected areas may act as refuges under future conditions and become important sources of genetic material to sustain coastlines not afforded the same level of protection.
